# Chemical signal diversity in male sand lizards (*Lacerta agilis*) along an urbanization gradient

**DOI:** 10.1038/s41598-025-90393-6

**Published:** 2025-02-26

**Authors:** Alejandro Ibáñez, Bartłomiej Zając, Izabella Sambak, Michał Woźniakiewicz, Aneta Woźniakiewicz, Maciej Pabijan

**Affiliations:** 1 Faculty of Biology and Environmental Protection, Department of Ecology and Vertebrate Zoology, University of Lodz, 90-237 Lodz, Poland; 2 Department of Comparative Anatomy, Institute of Zoology and Biomedical Research, Faculty of Biology, Jagiellonian University, Kraków, Poland; 3 Department of Analytical Chemistry, Laboratory for Forensic Chemistry, Faculty of Chemistry, Jagiellonian University, Krakow, Poland

**Keywords:** Femoral glands, Chemical communication, Habitat, Body condition, Lacertidae, Pheromone, Chemical ecology, Herpetology

## Abstract

Urban areas have globally expanded recently and will likely continue to do so in the near future. Although the impact of urbanization on acoustic and visual sexual signals has received considerable attention, other aspects, such as its influence on chemical signaling, remain poorly studied. Many lizard species possess femoral glands, i.e. prominent epidermal glands on the underside of the thighs producing a wide variety of compounds used in chemical signaling. Here we assessed the effect of urban, suburban and rural habitats and individual body condition on variation of chemical signal composition in the sand lizard (*Lacerta agilis*). By using gas chromatography coupled with mass spectrometry, we characterized chemical compounds present in secretions of lizard femoral glands. We found that lizards from suburban and urban habitats had the highest diversity of chemicals, while rural habitats showed significantly lower compound diversity. Lizards from urban and suburban habitats had high amounts of several compounds, including α-tocopherol, an antioxidant molecule that may counterbalance the damaging effects of irradiation on other pheromones. Chemical signals may not only depend on habitat characteristics but individual traits, such as body condition, may also affect chemical compound diversity. Body condition did not significantly differ across habitats, and we did not find an association between body condition and chemical composition of femoral gland secretions. We argue that environmental differences (more extreme in cities) as well as other factors such as increased stress may shape the amounts and diversity of semiochemicals in sand lizards. Our study provides insight on how environmental conditions imposed by urban–rural gradients may modulate chemical communication in vertebrates.

## Introduction

Urbanization has dramatically increased in recent times and will further expand in the twenty-first century^1^. By the year 2050, it is projected that more than two-thirds of the human population will be living in urban areas^2^. The urbanization of natural habitats has profoundly altered the landscape and has imposed substantial selective pressures on many organisms, with a wide range of outcomes^3,4^. Although urban land conversion is detrimental to many organisms^5^, some species have managed to adapt to and even flourish in urban environments^6^.

Urbanization can exert strong selection on animal signaling systems, coercing rapid changes in communicative signals^7,8^. For instance, urban birds are subjected to anthropogenic noise that can mask acoustic signals in urban habitats^9,10^. Birds living in cities need to cope with higher environmental noise than those from rural habitats and theoretical models predict a reduction in active space and vocal repertoire of bird species from urbanized areas^11^. Although there is increasing interest in how urbanization affects animal communication, most signaling channels and taxa remain poorly known^8^. For instance, most research on the effect of urbanization on sexual signaling has focused on acoustic and visual communication channels in birds^8^, however some modalities of communication (e.g. chemical signals) and taxonomic groups (e.g. reptiles) are clearly underrepresented. Therefore, more studies on the interplay between pheromones and urbanization are urgently needed to fully understand how animal mating systems evolve in rapidly expanding urban landscapes.

Chemical communication is arguably one of the most ancient ways of communication among animals and is involved in crucial activities such as identifying a suitable partner (species and sexual recognition), assessing mate quality, and avoiding predators^12^. Chemical signals are usually stringently connected to the habitat in which animals live, in large part due to the physical and chemical properties of the environment in which signals are emitted. Comparative analyses in mammals and reptiles have shown that semiochemical composition covaries with habitats and climatic variables^13,14^. In the context of urbanization, urban heat islands, through effects on microenvironmental parameters such as temperature and humidity^8^, may be particularly forceful in altering volatile pheromone-based signaling^15^. Stressful thermal conditions may affect pheromone synthesis, chemical signal durability after emission, as well as signal detectability^16^. For example, in the lacertid lizard *Iberolacerta cyreni* warmer temperatures inhibited the persistence of male chemical signals and impeded detectability by females^17^. In spiny-tailed lizards, *Uromastyx aegyptia microlepis*, semiochemical composition is fine-tuned to the microclimatic conditions inside the burrows in which this species resides^18^. Some compounds could be especially important in hot habitats since high temperatures might increase semiochemical fade out^13^. For instance, cholesterol, an abundant semiochemical in vertebrate epidermal secretions, is thought to stabilize other molecules in hot microhabitats^19,20^. Moreover, highly humid environments may lead to lipid oxidization. It has been hypothesized that some compounds such as α-tocopherol could protect other lipid compounds from oxidization in humid habitats^21^. In general, because chemical signals are tightly linked to their environment, it is expected that semiochemical composition is altered in urban–rural habitat gradients.

The sand lizard *Lacerta agilis* Linnaeus (1758) is a highly adaptable lacertid found in a broad range of habitats including agricultural landscapes, forests and forest edges, meadows, grasslands and shrublands, but also in anthropogenic habitats within city limits^22,23^. *Lacerta agilis* possesses epidermal glands (i.e. femoral glands, FGs) situated along the ventral surface of the hind limbs that are prominent during the spring breeding season. These organs produce secretions (semiochemicals) that are released to the external environment through pores. These secretions may be involved in intraspecific communication^24^ or mate assessment^25,26^. In the breeding season, male-to-male interactions including displays and combat are readily observable in sand lizards^22^ although the roles of FG secretions in determining male social status or for marking territories remain unexplored. In other lacertids, FG secretions may be important in species and mate recognition as well as agonistic interactions to establish dominance^27,28^.

Inter-individual variation in lipids and proteins secreted by sand lizard FGs^14,29^, as well as the capacity of this species to occupy diverse habitats including natural, rural and urban areas, make it an ideal model to test for effects of urbanization on chemical signaling. Here we determined the composition of the lipophilic fraction of FG secretions in male sand lizards from urban, suburban, and rural habitats. Given the more extreme conditions of urban habitats in terms of temperature and humidity^8,15,16^, we hypothesized that city-dwelling lizards would have a distinct chemical profile compared to lizards from non-urban environments while we predicted that suburban lizards would display an intermediate pattern between both habitats. We expected that constraints imposed by urban habitats affect chemical diversity and that some compounds (e.g. cholesterol and α-tocopherol) may vary sharply across environments as they could be relevant in stabilizing and protecting semiochemicals in more extreme environments.

Differences in resource availability, activity patterns or predatory regimes in cities^30^ affect the diet of city-dwelling individuals. In turn, dietary composition may have a strong influence on compounds used in chemical signaling due to changes in body condition of individuals and production or sequestration of specific biochemicals^31^. Thus, if urbanization influences chemical signaling indirectly through an effect on physiology then we would expect a correlation between semiochemical composition and body condition. For instance, habitat might affect lizard condition and in consequence modulate the capacity of individuals to produce chemical compounds. We therefore assessed whether the chemical profiles of male sand lizards depend upon their body condition. If body condition, chemical profile and habitat type co-vary in sand lizards, then an indirect effect of urbanization on chemical signaling would be supported. Conversely, no relation between body condition and chemical profile would indicate that direct physical factors outweigh the indirect effects of diet and body condition on semiochemical composition.

## Material and methods

### Study area and species

**Fig. 1:**
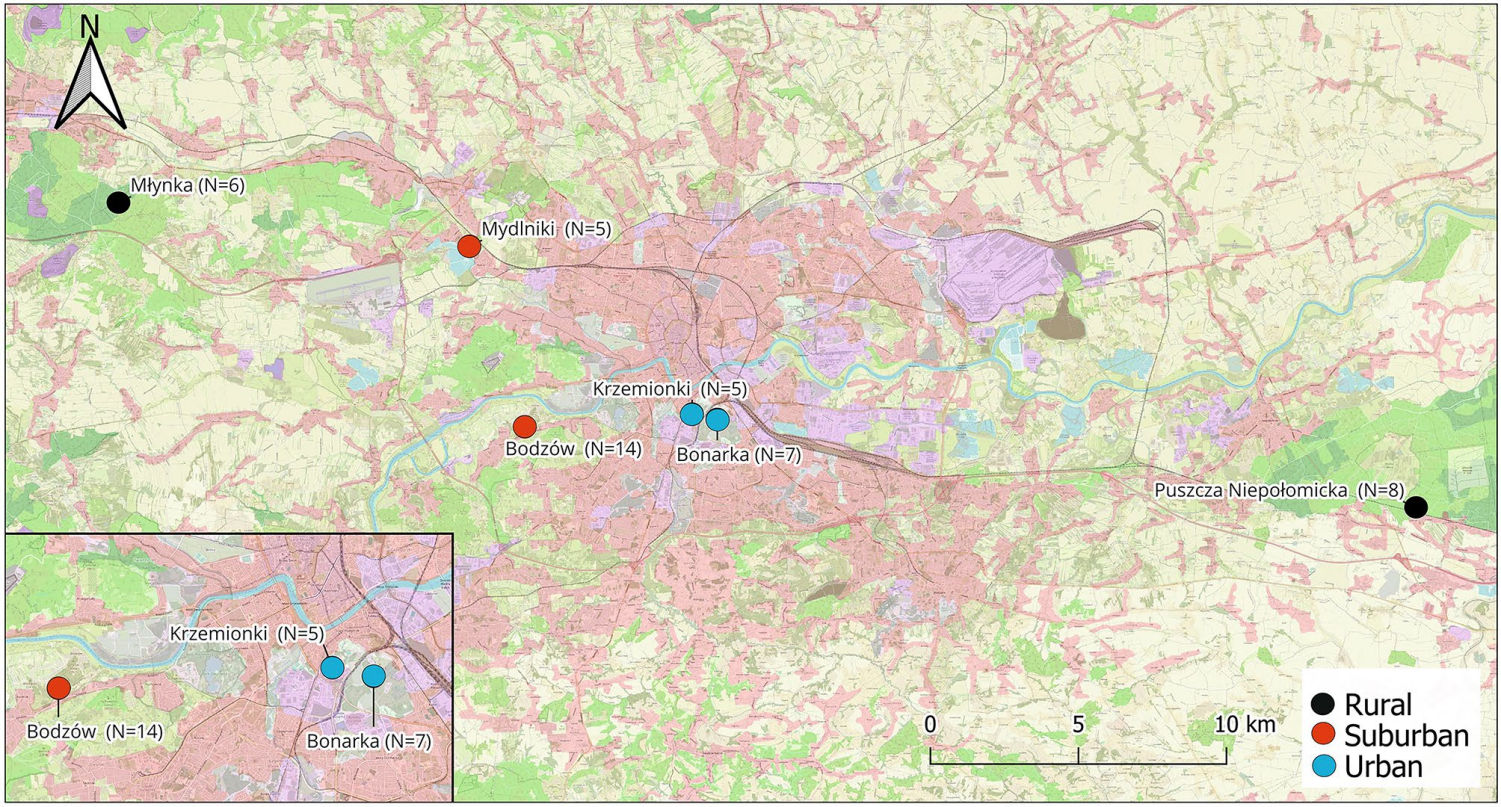
Map with the main sampling sites classified according to habitat urbanization.

Lizards were obtained from six localities located in the city of Krakow, southern Poland, and its vicinity. Krakow has a human population of around 800,000 and is highly urbanized but with small patches of suitable lizard habitat within the city such as abandoned limestone quarries. Here we classified each locality into one of the three habitat categories: “urban”, “suburban” and “rural”. This classification was based on three different parameters: 1) proximity to the city center of Krakow; 2) level of human footprint and 3) proportion of impervious surface. We extracted Global Human Footprint index (GHFI)^32^ categories at a spatial resolution of ~ 1 km (Table 1). The GHFI dataset was created from nine global data layers covering human population pressure (population density), human land use and infrastructure (built-up areas, nighttime lights, land use/land cover), and human access (coastlines, roads, railroads, navigable rivers)^32^. We calculated the average percentage of impervious surface from a high-resolution layer database of the European Environment Agency (https://land.copernicus.eu/pan-european) in a 1 km buffer around the central point of each study site using Quantum-Gis 3.28.6 (function “Zonal Statistics”) as in ^33^. The two urban localities (~ 3 km from the center) were considered to contain independent lizard populations despite geographical proximity due to an inhospitable built-up area (including a busy road) between them (Fig. 1). Both localities show high levels of human footprint and imperviousness (Table 1). Two further localities were classified as suburban as they were located within the city of Krakow but farther from the center (6 and 7.5 km, Fig. 1) with intermediate to high human footprint and intermediate values of imperviousness (see Table 1). Rural habitats were located > 15 km away from the Krakow city center (2 sites; Fig. 1) and had low levels of human footprint and reduced impervious index (Table 1).

**Table 1:** Overview of the samples of FG secretions from sand lizards collected at each site and habitat type used in this study. Global Human Footprint index (Human Footprint) categories at a spatial resolution of ~ 1 km are provided for each locality. Percentage of impervious surface in 1 km buffer around central point of each study site. Distance to the city center (in Km) and sample size (N) per each locality are shown.

Locality	Habitat	Human Footprint (%)	Imperviousness (%)	Distance (Km)	N
Młynka	Rural	30–40	0.3	19.3	6
Puszcza Niepołomicka	Rural	30–40	2.2	25.6	8
Bodzów	Suburban	60–80	4.6	6	14
Mydlniki	Suburban	60–80 / 80–100	12.4	7.5	5
Bonarka	Urban	80–100	24	3.2	7
Krzemionki	Urban	80–100	34.3	2.75	5

A total of 45 male sand lizards were collected during the mating seasons (April-July) of 2019 and 2021. Only FG secretions of male lizards were considered in this study since these glands are dimorphic and more developed in males than females^34^. Lizards were sexed according to external features (presence of nuptial green coloration and prominent FGs). After sampling, lizards were measured with a caliper (Snout-Vent Length = SVL) or in some instances their SVL was estimated from photographs with a millimetric scale. These methodologies yield equivalent estimates^35^. We used a pesola or an electronic balance to weigh the lizards. Morphometric measurements were taken from 42 individuals. After sampling and measuring all lizards were released at their sites of capture.

### Sampling and chemical analysis

Femoral gland secretions were obtained from 45 individuals by gently squeezing the femoral pores. The secretions were stored in glass vials with inserts previously filled with dichloromethane. Vials were closed with silicone/PTFE screw caps and stored in cold conditions (approx. − 20 °C) until gas chromatography coupled with mass spectrometry (GC/MS) analysis. Although we did not measure or weigh the amount of secretion from each lizard, the collection procedure was standardized and ensured roughly similar amounts per individual. Samples from all individuals were used for the analysis of the lipophilic fraction of femoral gland secretions.

Before GC–MS analysis, all samples were subjected to a derivatization protocol to introduce a trimethylsilyl functional group to the compound of interest. The derivatization process used here was intended to improve the detection and identification of chemical compounds in lizard samples. The derivatization protocol included multiple steps. First, samples were warmed to ambient temperature and dichloromethane was evaporated to dryness under a stream of nitrogen at 40 °C. Then, 10 μL of acetonitrile and 50 μL of 99% N,O-bis(trimethylsilyl)trifluoroacetamide (BSTFA) with 1% trimethylchlorosilane (TMCS) mixture were added into the dry residue. The amber glass vials were tightly closed and the derivatization process was carried out at 60 °C for 1 h. Afterwards, the vials were opened and the derivatization solution was evaporated under a stream of nitrogen at 60 °C and the dry residue was dissolved in 25 μL of dichloromethane. Three consecutive extractions were performed for every sample, and the separated organic solvents were put into one vial. The collected solvent was evaporated to dryness under a stream of nitrogen at 40 °C and the dry residue was subjected to the derivatization procedure described above.

A GC–MS system consisting of a 6850 Series II gas chromatograph and a 5975C MSD mass spectrometer (Agilent Technologies, Santa Clara, CA, USA) equipped with a HP-5 ms capillary column (30 m long, 0.25 mm i.d., 0.25 μm film thickness, Agilent Technologies, Santa Clara, CA, USA) was used for analyzing all samples. The oven temperature program was set up to hold 50 °C for 10 min, then the temperature was ramped up to 280 °C with a rate of 5 °C/min, and then held for 30 min. Helium (5.0) was used as a carrier gas with a flow rate of 1.0 mL/min. Splitless injection of two μL was performed at injection port heated to 280 °C. The MS transfer line temperature was set to 280 °C and ion source temperature to 230 °C. EI source operated at 70 eV, and the mass range for the MS detector in scan mode was from 39 to 400 m/z (from 5 min) and from 39 to 600 m/z (from 20 min). The identification of all detected compounds was based on a semi-automatic library search (all results were inspected by the operator) with the NIST 11 database (NIST, Gaithersburg, MA, USA).

Compounds were tentatively identified on the basis of their mass spectra match and the retention times of detected peaks were additionally used to compare samples. Data was filtered in several steps. First, we excluded unidentified compounds. We considered a compound unidentified if it was unmatched or if it had a match lower than 850. However, an exception was made for some sample compounds with a match value below 850. Namely, in such rare cases, identification was based on retention time and similar mass spectrum to a compound with match over 850 at this same retention time in other samples; all compounds with a very low match were excluded. Afterwards, we filtered out compounds that appeared in only one sample, i.e., only compounds appearing in at least two samples were retained^36^. As a general rule, we filtered any compound suspected to be non-naturally occurring in FGs. For instance, we found phthalates that are typically present in plastics and other artificial material^37^ that we considered as compounds of foreign (i.e. non-lizard) origin. Control blank tubes previously filled with dichloromethane and treated in the same way as samples were used to further exclude compounds that could have been introduced during the sampling procedure. Compounds naturally occurring in FGs were also filtered if they were present in at least two control tubes (e.g. hexadecanoic acid, trimethylsilyl ester). An exception was made for 13-Docosenamide, (Z)- which occurred in one control but was nonetheless excluded because it was present in several FG secretion samples. The strict filtering of compounds imposed in this study resulted in a conservative but reliable dataset on natural compounds originating from sand lizard FGs.

As previously described, a derivatization process was carried out for all samples to increase the detectability and stability of compounds. However, TMS derivatives could occur together with their parent compounds leading to statistical non-independence. To resolve this issue, we only considered trimethylsilyl (TMS) derivatives for most compounds for statistical analysis, and thus, non-derived compounds were filtered out. For instance, tocopherols, fatty acids, alcohols and steroids were only considered in TMS forms and any compound from these classes that was not TMS derived was excluded. However, compound classes that are resistant to the derivatization process were considered in their parent form (e.g. alkanes and alkenes).

We normalized the peak areas by dividing the area of a given compound by the area of a compound present in all samples (e.g. similar to an “internal standard”). The contaminant phthalic acid, hept-4-yl isobutyl ester was chosen for area normalization as it was present in all samples and controls. This compound likely originated from the dichloromethane bottle sealing used during sampling, and thus, we assumed similar amounts were present. Normalized peak ratios were used for further analysis. This method has the advantage of not being affected by the number of peaks present in a sample.

### Statistical analyses

We computed the Shannon diversity index using the normalized peak ratio values (see below for a summary of all R packages used). Body condition index (BCI) was calculated as the residuals between log transformed values of body mass and SVL. Morphometric measurements were missing for three individuals, therefore, a total of 42 lizards were included for BCI calculation. We used linear mixed-effects models to test for differences across habitats (urban, suburban and rural) on BCI and chemical diversity (Shannon index), including habitat as a factor and locality as a random effect due to the hierarchical structure of our data. The statistical significance of the fixed factor (habitat) was tested by estimating F values and their associated p values. In addition, we fitted models without habitat (“null models”) and compared these to the “full model” (i.e., models with habitat as a fixed factor) by using likelihood ratio tests (L-ratio). Pairwise contrasts were carried out to examine which habitat factor levels differed significantly. Furthermore, we carried out an ANCOVA to test the effect of habitat on chemical diversity adding BCI as well as the interaction of habitat and BCI as covariates.

We used a PERMANOVA to examine how habitat and locality contribute to variation in overall similarity or dissimilarity of chemical composition^38^. This method offers the possibility to test for between-group differences and can incorporate multiple variables, i.e. we were able to include all compounds in the same model instead of calculating multiple univariate models. As input we used logarithmically transformed [natural log (x + 1)] normalized peak ratio values for all compounds. Habitat and locality were tested sequentially in this order and when statistical differences were detected in each factor, we carried out multilevel pairwise comparisons to determine which pair levels differed. We also visualized the results with non-metric multidimensional scaling (NMDS). A distance matrix on log transformed peak ratios was used as input. To examine any potential temporal effect originating from our sampling strategy, we carried out a NMDS plot excluding the samples collected in the year 2021 (i.e. six samples from rural habitat). The resulting plot showed a similar pattern to the plot incorporating data from both 2019 and 2021 (see supplementary Fig. S1 and below). An NMDS with locality as a factor was also carried out to examine the distribution of samples across this parameter (supplementary Fig. S2).

In addition to PERMANOVA, we also used a nested PERMANOVA to examine the effect that locality nestedness on habitat might have on chemical dissimilarity.

We used Random Forest Classification (RF) to identify predictive lipids that could contribute to habitat differentiation of chemical profiles (using log transformed normalized peak ratios). We selected the most influential compounds contributing to habitat grouping for further linear mixed models including habitat as a factor and locality as a random term as described before. Pairwise comparisons were carried out to discern statistical differences across habitat types.

Residuals of the models were visually assessed (residuals vs. fitted values plot and normal Q-Q plot) showing that assumptions were met for most models. Linear mixed-effects models are most pertinent for hierarchical datasets and are robust to violations from distributional assumptions being usually more powerful than alternative analyses^39^.

Spearman correlations were used to test whether amounts (log normalized peak ratios) of the five most influential compounds were related to BCI.

Most of the analyses were carried out with R software using the following packages: vegan^40^, pairwiseAdonis^41^, nmle^42^, multicomp^43^ and BiodiversityR^44^. RF analysis was carried out using the web interface MetaboAnalyst 6.0.

## Results

### Chemical compounds of sand lizard femoral gland secretions

Taking into account 45 samples used in this study and after strict filtering, a total of 31 compounds were included in our database for further analysis (see supplementary Table S1). Most of the compounds considered here are TMS derived. In the following we use simplified names by omitting “trimethylsilyl” or its acronym (see supplementary Table S1 for an overview of all compounds with full names).

The main classes of compounds were steroids (summation of normalized peak ratio means: 39.5), followed by tocopherols (17.9) and carboxylic acids (9.6). Small amounts of other compound classes including alcohols, alkanes, alkenes, carbohydrates and one inorganic acid were also found. Cholesterol and α-tocopherol were the two most abundant compounds constituting the bulk of lizard FG secretions (mean of normalized peak ratios 21.9 and 17.9 respectively; see supplementary Table S1). Further abundant compounds in FG secretions included tetradecanoic acid (peak ratio: 7) and campesterol (peak ratio: 5.3; Table S1).

### Chemical profiles, body condition and habitat urbanization

**Fig. 2:**
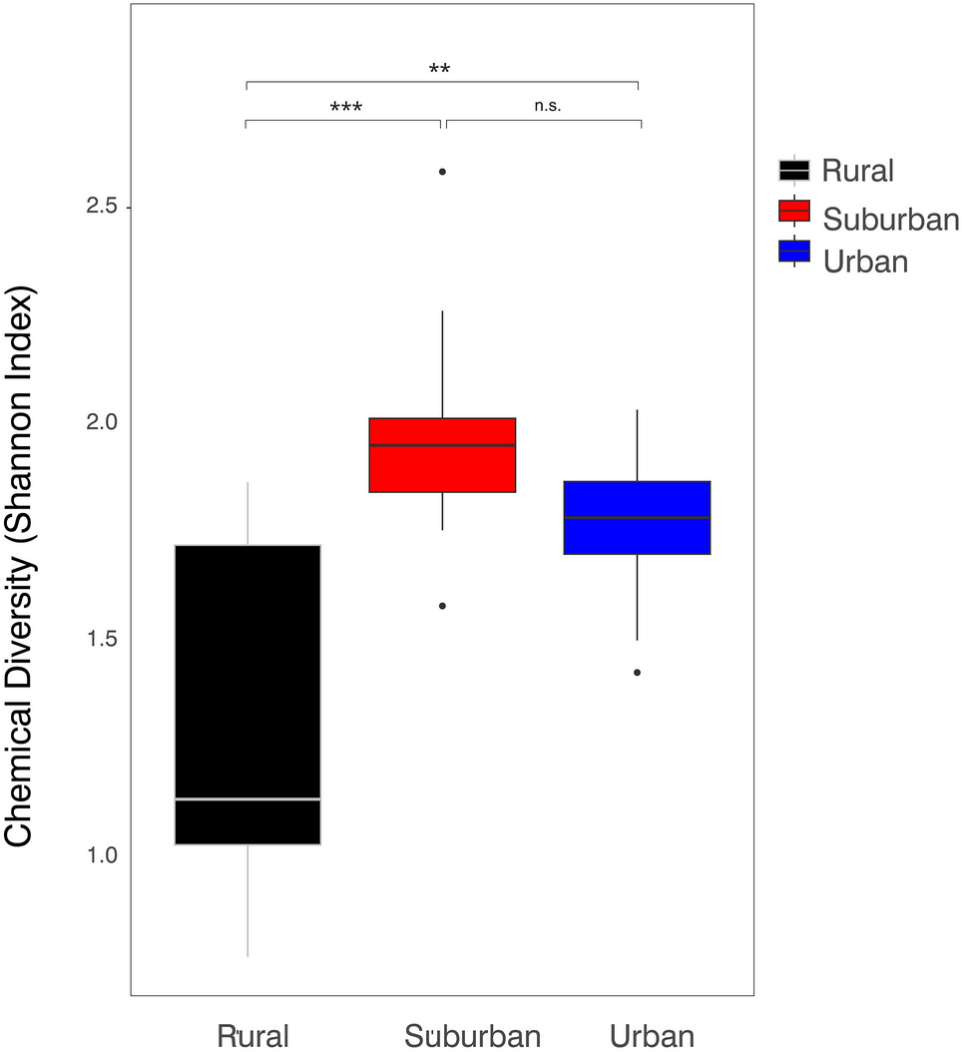
Boxplot showing chemical diversity (Shannon Index) across habitats. Significant pairwise comparisons are marked with asterisks.

Shannon index (chemical diversity) ranged from 0.77 to 2.58 (mean ± SD = 1.7 ± 0.39). A linear mixed-effect model revealed that Shannon index differed between habitats (F_2,3_ = 10.16, P = 0.046; see Fig. 2). Similarly, the model with habitat as a fixed factor had a significantly better fit than the null model (see supplementary Table S2). Urban and suburban lizards had a higher chemical diversity (calculated as Shannon index) compared to lizards from rural habitats (pairwise contrasts; both P < 0.01; see Fig. 2). However, chemical diversity did not differ significantly between lizards from urban and suburban habitats (P = 0.52). Body condition index did not significantly change across habitats (linear mixed-effects model; F_2,3_ = 2.03, P = 0.277), although we found considerable variation across localities (see supplementary Figure S3). In line with this result, a likelihood test revealed that models for BCI with and without habitat as a fixed factor did not differ (see supplementary Table S2). Moreover, including BCI as a covariate in an ANCOVA model showed that the effect of BCI on chemical diversity (P = 0.466) was insignificant, as was the interaction between habitat and BCI (P = 0.196) while the effect of habitat type remained significant (P < 0.001).

**Fig. 3:**
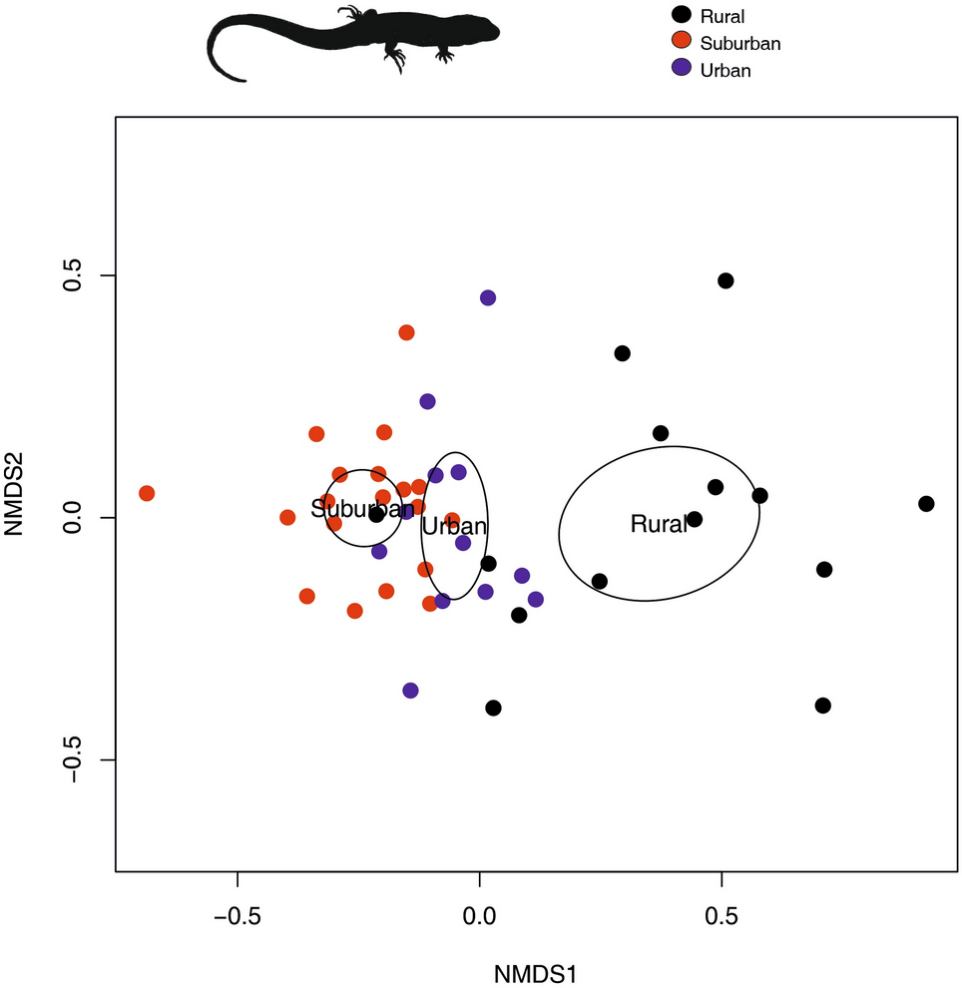
Visualization for 45 samples of lizards using Nonmetric Multidimensional Scaling (NMDS) based on a distance matrix of log transformed normalized peak ratios. The ordination plot includes 95% confidence ellipses around species centroids. Each color represents one habitat type (black: rural; red: suburban; blue: urban). For animal silhouette credits see acknowledgements.

There was notable variation in overall chemical composition (log transformed normalized ratios) across habitat types (Fig. 3) and localities (supplementary Fig. S2) as displayed in NMDS plots. This pattern was similar if samples from the year 2021 were excluded (see supplementary Fig. S1), although differences were more obvious when incorporating all samples (Fig. 3). A PERMANOVA model showed an effect of habitat type (F = 12.16; P = 0.001) and locality (F = 2.75; P = 0.005) but more variation was explained by the former (R-squared = 0.34 and 0.12, respectively). However, the difference across habitats could be due to a heterogeneous dispersion between groups (betadisper, P = 0.003; see Fig. 3). Pairwise comparisons of chemical composition for habitat combinations (urban-suburban, rural–urban, rural-suburban) were significantly different (Table 2). Multiple differences across some localities were revealed (see supplementary Table S3). However, when using nested PERMANOVA only locality was significant (F = 2.75; P = 0.004) while habitat showed a non-significant trend (F = 4.43; P = 0.06). This suggests a relatively strong effect of locality nested within habitat when considering overall chemical composition.

**Table 2:** Habitat multilevel pairwise comparisons to test for differences in chemical composition across habitat types (i.e. Urban, Suburban and Rural). Log transformed normalized peak ratio values were used as input. Multilevel pairwise-comparison adjusted P values are shown (p.adjust = ‘fdr’).

Habitat pairs	Df	Sums Of Sqs	F.Model	R2	p.value	p.adjusted
*Suburban vs Urban*	1	0.143	4.462	0.1333	0.003	0.003
*Suburban vs Rural*	1	0.829	17.641	0.363	0.001	0.0015
*Urban vs Rural*	1	0.401	7.646	0.242	0.001	0.0015

**Fig. 4:**
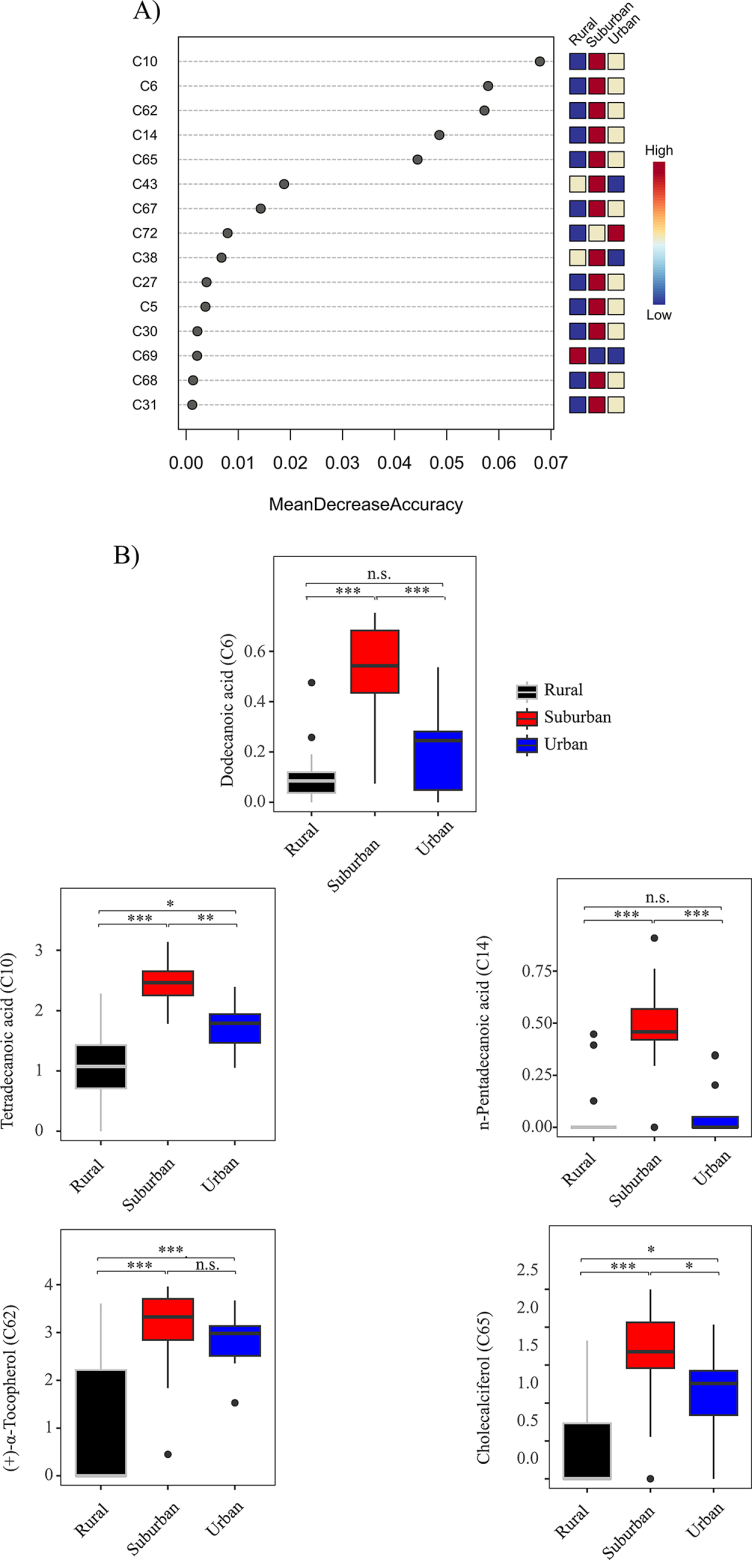
(**A**) Random forest variable importance plot based on log transformed normalized peak ratios. Mean decrease accuracy is the measure of the performance of the model without each compound. A higher value indicates the importance of that metabolite in predicting group (urban, suburban, and rural). Removal of that metabolite causes the model to lose predictive accuracy. Compound names are shown in supplementary Table S1. (**B**) Box plots of the top five compounds from random forest analysis. Amounts (log peak ratios) for each habitat are shown. Significant pairwise comparisons are marked with asterisks.

Random Forest classification revealed compounds that had a prevailing effect on habitat separation (Fig. 4A). Habitat-relevant compounds included dodecanoic acid (C6; compound numbers as in supplementary Table S1), tetradecanoic acid (C10), n-pentadecanoic acid (C14), α-tocopherol (C62) and cholecalciferol (C65). Linear mixed-effects models were carried out on the top five compounds. These analyses revealed a significant effect of habitat for all top five compounds (see Table 3; Fig. 4B). Similarly, models including habitat as a fixed factor had a better fit when compared with null models for all five compounds (supplementary Table S2). Pairwise comparisons revealed significant changes across habitat levels. For example, the level of α-tocopherol was significantly higher in both urban and suburban lizards compared to rural ones (P < 0.05), but no difference was detected between urban and suburban habitats (P = 0.81)—see Table 4 for all pairwise comparisons.

**Table 3:** Linear mixed-effects models testing the effect of habitat (models included locality as a random factor) on the top five chemical compounds contributing to habitat differences according to Random Forest analysis (see Fig. 4). Log normalized peak ratio areas used as input. Numerator degrees of freedom (numDF) and denominator degrees of freedom (denDF) are shown.

*Compound*	*numDF*	*denDF*	*F*	*P*
Dodecanoic acid (C6)	2	3	20.73	0.018
Tetradecanoic acid (C10)	2	3	19.03	0.020
n-Pentadecanoic acid (C14)	2	3	12.38	0.036
( +)-α-Tocopherol (C62)	2	3	16.12	0.025
Cholecalciferol (C65)	2	3	16.86	0.023

**Table 4:** Multiple comparisons of means using Tukey contrasts and single-step method for adjusting *P-*values using the ‘glht’ function from the R package ‘multcomp’. P-values for each comparison are shown for top five compounds across habitat types. Log normalized peak ratios were used as input.

*Habitat*	*Dodecanoic acid*	*Tetradecanoic acid*	*n-Pentadecanoic acid*	*(* +*)-α-Tocopherol*	*Cholecalciferol*
Suburban-Rural	< 0.001	< 0.001	< 0.001	< 0.001	< 0.001
Urban–Rural	0.429	0.027	0.999	< 0.001	0.019
Urban-Suburban	< 0.001	0.002	< 0.001	0.81	0.021

**Fig. 5:**
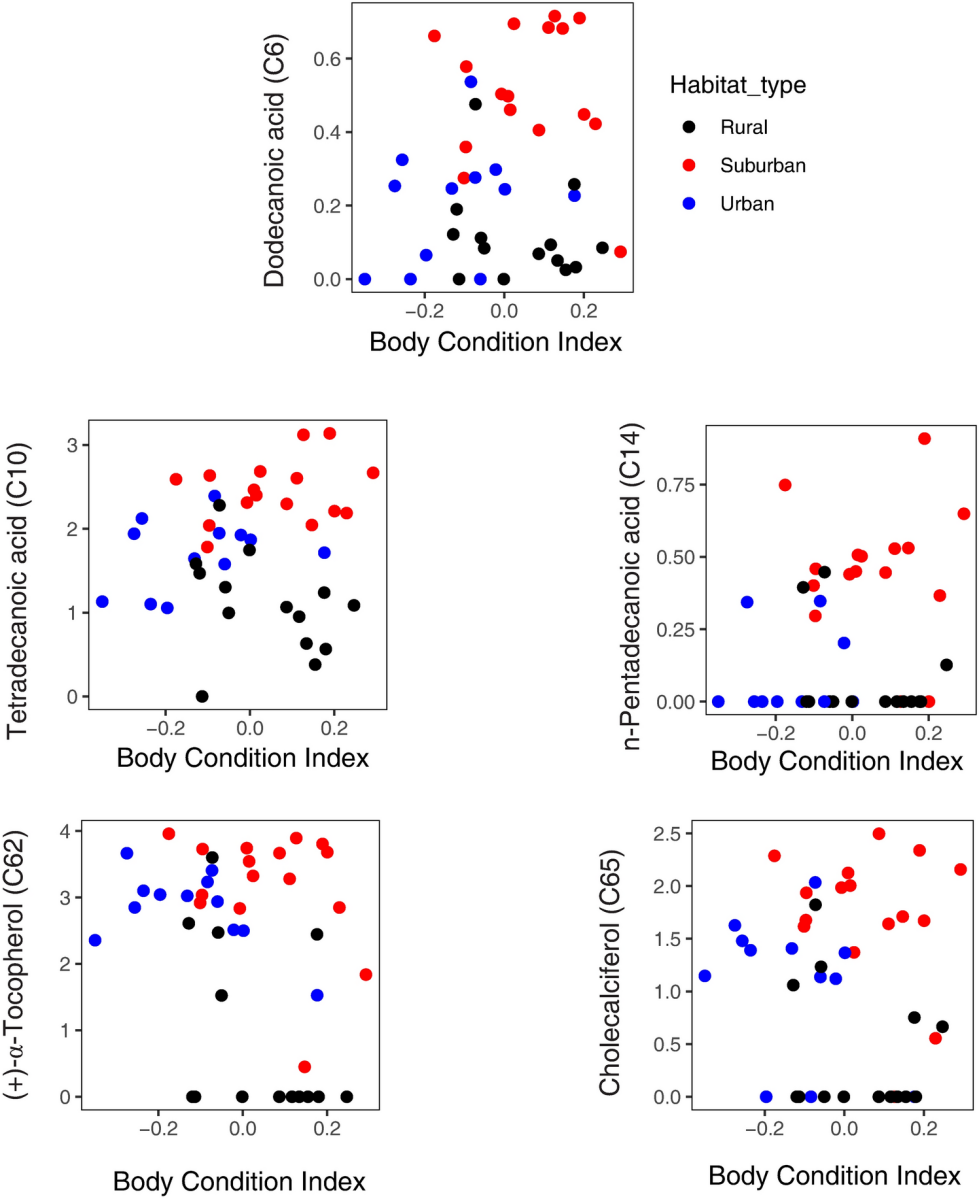
Scatterplots for the amounts (log peak ratios) of top five compounds and body condition index per each habitat. None of the correlations were significant (P > 0.05; see supplementary Table S4).

The amounts of the five most influential compounds were not significantly correlated to BCI. Correlations were carried out separately in each habitat type and revealed insignificant relationships between these parameters (all P > 0.05; supplementary Table S4). Scatterplots for the five compounds are shown in Fig. 5.

## Discussion

This study revealed that the chemical composition of femoral gland (FG) secretions depends on the habitat experienced by individual male sand lizards. Our results suggest that the level of habitat urbanization influences both chemical diversity and composition. We found that suburban and urban lizards have a higher chemical diversity than lizards from rural habitats. Moreover, habitat differences were driven by a subset of compounds, particularly α-tocopherol, that was found in larger amounts in suburban and urban environments compared to rural ones. Our linear mixed-effects model indicated that body condition did not significantly differ across habitats. Body condition seemed to be uncoupled from FG chemical diversity or relative amounts of habitat-related compounds. In terms of an urbanization gradient, we found the highest chemical diversity and highest abundances of major compounds in FG secretions in lizards from suburban habitat, suggesting a non-linear relationship between urbanization and FG chemical profile in sand lizards. We suggest that habitat heterogeneity or complexity (highest in suburban areas) may be contributing to the differences in chemical composition.

### Chemicals, body condition and habitat urbanization

Our research revealed that the lipophilic compounds released by FGs vary across habitats with distinct degrees of anthropogenic pressure. In our study, rural habitats were restricted to forested areas with small meadows offering relatively uniform ecosystems with low human impact. In contrast, urban and suburban habitats offered structurally complex environments with both natural and manmade elements (such as, e.g., abandoned limestone quarries), as well as a wide variety of microhabitats ranging from rocky surfaces through grassland and shrubland to woodlands. In addition, the urban habitats were situated within a city of more than 800,000 residents (see Fig. 1), and therefore, were exposed to artificial stressors as well as high levels of human activity that might affect the physiological condition of lizards.

Chemical signal makeup in lizards is strongly influenced by environmental factors^14,45,46^, sexual selection^47,48^, and neutral population genetic processes^49^. We found that sand lizards from urban and suburban habitats have a higher chemical diversity and different chemical composition of FG secretions compared to lizards from rural forest habitats indicating a potential phenotypic response of lizards to local environmental conditions aimed at maximizing semiochemical efficiency. Both humidity and temperature may affect semiochemical persistence in the environment^13,50^. The suburban and urban localities studied herein are part of an urban heat island and are relatively warmer when compared to rural areas^51^, and this effect was particularly clear at urban sites^52^. We hypothesize that lizards living in urban areas may mitigate the degradation of lipophilic compounds due to high temperatures^53^ (or other related factors such as UV-irradiation) by producing a more diverse array of compounds in FG secretions, especially those acting as pheromone-protectors. Alternatively, high chemical diversity of FG secretions in urban and suburban lizards could be related to elevated metabolic rates driven by environmental factors such as temperature^54,55^. Phenotypic plasticity may directly impact semiochemical composition. For instance, in European wall lizards (*Podarcis muralis*)*,* FG composition changes according to the thermal environment, enhancing signaling efficacy under warm conditions to preclude the fade-out of femoral secretions^56^. Our results are in line with ^56^, indicating that lizards may modify their chemical profiles as a function of habitat-related conditions (e.g. temperature, humidity). As pheromones are directly related to mating opportunities, it is expected that environment-related variation in semiochemicals may consequently affect individual fitness^57^. Alternatively, a possible scenario could be that warm environments increase metabolism^54,55^, leading to an increased production of metabolic by-products that are not directly linked to communicative processes.

Sand lizard body condition did not vary significantly across habitats, although differences in BCI among localities were sometimes substantial (supplementary Fig. S3). For instance, most lizards at one of the urban sites (Krzemionki) had low and negative BCI values, while lizards in suburban (Bodzów) and natural (Młynka) sites had high BCIs. These results suggest that food resources (e.g. arthropod abundance^58^) differ across the studied localities, or that other factors such as conspecific or predator abundance^59^, could be influencing lizard BCI. Similarly, a quantitative metanalysis found no effect of urbanization on body condition in lizards^30^. Importantly, our study did not detect a statistical relationship between chemical diversity of FG secretions and BCI nor between the interaction of habitat and BCI. Therefore, our results indicate that variation in BCI did not reflect FG chemical composition, suggesting that different factors modulate these two phenotypic traits.

### Habitat-related compounds: pheromones or chemical-protectors?

Although behavioral tests are needed to infer the functions of the compounds detected in FG secretions, previous work allows for some tentative hypotheses. Fatty acids are among the main compounds in many reptilian epidermal glands, including those of lacertid lizards (reviewed by Weldon and collaborators^20^). The fatty acids found in FG secretions of lizards are an important source of energy, contributing to the replenishment of fat stores and many physiological processes^60^. Allocating these compounds to FGs for semiochemical production may therefore entail costs and be condition-dependent^61^. In some species, fatty acids are used to convey reliable information to potential partners. For instance, male rock lizards *Iberolacerta cyreni* may release high amounts of oleic acid to attract females and thus increase their reproductive success^28^. In our study, sand lizards from suburban habitats had large amounts of three fatty acids (dodecanoic acid, tetradecanoic acid, and n-pentadecanoic acid) but this was not related to individual BCI. We hypothesize that the secretion of these compounds may have an environmental context, although the underlying mechanisms leading to this variation remain unknown.

Besides fatty acids, FG secretions from suburban lizards were rich in α-tocopherol (vitamin E) and cholecalciferol (vitamin D3). These compounds, or similar ones such as provitamin D, may function as sexual signals in other lacertid lizards. For instance, experiments with Iberian lizards (genus *Podarcis*) showed that dietary supplementation of vitamin D or vitamin E increased the attractiveness of potential partners^62,63^. However, our results indicate that variation in these compounds could be related to the more extreme conditions experienced by lizards in anthropogenically perturbed habitats.

One of our expectations was that cholesterol—a compound that has been postulated as an unreactive matrix protecting other chemicals in FG secretions^19,20,64^—could be more prominent in urban habitats because of their warm temperatures. We did not find evidence to support this hypothesis as cholesterol was not among the compounds detected in the RF analysis (Fig. 4). However, cholesterol was a major compound in FG secretions irrespective of habitat and relevant in terms of its share with respect to other analytes. However, the amounts of another major compound (α-tocopherol) differed across habitats. Although tocopherol is involved in chemical signaling in some lacertids (see above), it has also been hypothesized to have a protective role against lipid oxidation. Gabirot and collaborators^21^ suggested that the large amounts of α-tocopherol found in *Zootoca vivipara* in humid habitats could protect other semiochemicals found in lizard secretions. This argument is based on the antioxidant activity of tocopherol that could inhibit lipid oxidation under wet conditions. We found a high amount of α-tocopherol in sand lizards from urban and suburban areas compared to rural (forested) ecosystems, suggesting that factors other than humidity are operating. For instance, ultraviolet (UV) irradiance can accelerate lipid degradation^65,66^, and α-tocopherol may have a protective role by preventing lipid oxidation in FGs^21^. It has been shown that tree height and canopy cover can substantially reduce solar irradiation^67^. Indeed, lizards living within Krakow (urban and suburban localities) likely receive intense UV-light radiation because of diminished tree cover (reflected by a higher impervious index). In contrast, sand lizards from rural habitats collected in this study originated from forested areas. In this context, the production of tocopherols could be less important for lizards in rural forested areas. A possible scenario is that both urban and suburban habitats may vary in one or more parameters (e.g. higher UV radiation) enhancing the production of α-tocopherol that would protect lipophilic compounds from oxidative degradation under these microclimatic conditions. Previous research has shown a tight link between environmental conditions, metabolism and oxidative-stress^68–70^. Urban and suburban habitat may present environmental challenges (e.g. heat, dryness and/or high solar radiation) that may directly affect the production of reactive oxygen species (ROS). Under these conditions antioxidant molecules such as tocopherols could have a pivotal role as cell protectors^71^ in city-dwelling lizards. In addition, cholecalciferol was higher in suburban lizards compared to both rural and urban lizards. The role of cholecalciferol in lizard secretions is unknown, but in other vertebrates it has an important role in maintaining proper functioning of the immune system^72,73^ and may counteract the negative effects of oxidative stress at the cellular level^74^. Suburban lizards may compensate for high stress by releasing cholecalciferol that could boost the immune response against stressors. However, this hypothesis does not explain the lower amounts of this compound in urban lizards.

### Limitations of the study

An important caveat of this work is the lack of environmental data including microhabitat availability for the studied localities. Although we have used three surrogate indices (distance to urban center, human footprint and imperviousness) to broadly capture environmental differences across an urbanization gradient, we acknowledge that the collection of data such as temperature and humidity (e.g. through dataloggers) of microhabitats used by lizards could substantiate our hypotheses on the factors determining the composition and diversity of chemical signals in urban and suburban lizards. Other uncontrolled factors such as exposure to wind, refuge availability, diet, vegetation and substrate type, conspecific density or predator pressure could also influence FG chemistry and should be explored in more detail. Moreover, temporal variation in semiochemical composition of FG secretions should be considered as a potential confounding factor of our study, although we note that we minimized this effect by restricting our sampling exclusively to the mating season of sand lizards^75–77^ when FG secretory activity is at a maximum^78,79^. This approach is similar to other studies testing the effect of environmental habitat conditions on chemical composition of lizard FGs (e.g. ^14,80^). In addition, future studies should examine the effect of habitat variation on other compounds such as proteins, since these are prevalent in sand lizard FGs^29^. Finally, replicated urban–rural sampling of lizards inhabiting other cities across the broad range of this species could test if there is a parallel response of FG semiochemical composition to urbanization.

## Conclusions

The results of our study revealed an effect of habitat and locality on lizard chemical diversity and composition. The effect of habitat was strong when considering chemical diversity and the amounts of the five most influential compounds. In addition, locality (nested in habitat) was also an important factor when examining overall chemical composition. Environmental conditions might be shaping the semiochemical profiles of lizards to a greater extent than factors such physiological condition of lizards, as we have not found an association between body condition and semiochemical diversity. Our results indicate an increase in chemical diversity, as well as higher relative amounts of pheromone-protector compounds (e.g. α-tocopherol) in urban and suburban lizards with respect to rural populations in forested areas. We conclude that the harsh conditions in urban and suburban areas may entail changes in the chemical signaling system of sand lizards. Although the reproductive consequences of these changes are unknown, our study provides the framework for further research examining the fitness-related effects of chemical signal variation across urban–rural gradients.

## Supplementary Information

Supplementary Information.

## Data Availability

The data underlying this article will be shared on reasonable request to the corresponding author.
