# Supplementary material for: Chemical signal diversity in male sand lizards (Lacerta agilis) along an urbanization gradient
Source: Sci Rep. 2025 Feb 26;15:6958. doi: 10.1038/s41598-025-90393-6 (PMC11865607; doi:10.1038/s41598-025-90393-6)

*Supplementary Material for: “****Chemical signal diversity in male sand lizards (Lacerta agilis) along an urbanization gradient****”*

Alejandro Ibáñez​^1*^, Bartłomiej Zając^2^, Izabella Sambak^2^, Michał Woźniakiewicz^3^, Aneta Woźniakiewicz^3^, Maciej Pabijan^2^

1-University of Lodz, Faculty of Biology and Environmental Protection, Department of Ecology and Vertebrate Zoology, Lodz, Poland

2-Department of Comparative Anatomy, Institute of Zoology and Biomedical Research, Faculty of Biology, Jagiellonian University, Kraków, Poland

3-Department of Analytical Chemistry, Laboratory for Forensic Chemistry, Faculty of Chemistry, Jagiellonian University, Krakow, Poland

*Corresponding author. University of Lodz, Faculty of Biology and Environmental Protection, Department of Ecology and Vertebrate Zoology, 90-237 Lodz, Poland.

Email: [alejandro.ibanez@biol.uni.lodz.pl](mailto:alejandro.ibanez@biol.uni.lodz.pl)

**Supplementary tables:**

Supplementary Table S1. Normalized peak ratios (mean and SD) for each compound characterized in sand lizard femoral gland secretions for each habitat separately and across all samples. Compound id’s are indicated in brackets.

| **Name (id)** | **Parent compound** | **Rural**  **(N=14)** | |  | **Suburban (N=19)** | |  | **Urban (N=12)** | |  | | **Total**  **(N=45)** | | |
| --- | --- | --- | --- | --- | --- | --- | --- | --- | --- | --- | --- | --- | --- | --- |
|  |  | *mean* | *sd* |  | *mean* | *sd* |  | *mean* | *sd* | |  | | *mean* | *sd* |
| Phosphoric acid, tris-TMS (C2) | Inorganic acid | 0.4 | 0.85 |  | 0.49 | 0.86 |  | 0.31 | 0.54 | |  | | 0.41 | 0.77 |
| Glycerol, tris-TMS (C3) | Alcohol | 0.02 | 0.09 |  | 0.03 | 0.13 |  | 0 | 0 | |  | | 0.02 | 0.1 |
| Tetradecanal (C5) | Alkane | 0 | 0.01 |  | 0.06 | 0.08 |  | 0.01 | 0.04 | |  | | 0.03 | 0.06 |
| Dodecanoic acid, trimethylsilyl ester (C6) | Carboxylic acid | 0.13 | 0.16 |  | 0.73 | 0.29 |  | 0.24 | 0.21 | |  | | 0.41 | 0.36 |
| Pentadecanal- (C7) | Alkane | 0 | 0.01 |  | 0.04 | 0.07 |  | 0.02 | 0.04 | |  | | 0.02 | 0.05 |
| Tetradecanoic acid, trimethylsilyl ester (C10) | Carboxylic acid | 2.51 | 2.24 |  | 11.52 | 4.59 |  | 4.93 | 2.37 | |  | | 6.96 | 5.28 |
| Carbohydrate Unidentified 1 (C12) | Sugar | 0 | 0 |  | 0.04 | 0.09 |  | 0 | 0 | |  | | 0.02 | 0.06 |
| n-Pentadecanoic acid, trimethylsilyl ester (C14) | Carboxylic acid | 0.08 | 0.19 |  | 0.65 | 0.36 |  | 0.09 | 0.16 | |  | | 0.32 | 0.39 |
| Carbohydrate Unidentified 4 (C17) | Sugar | 0 | 0 |  | 0.03 | 0.07 |  | 0.01 | 0.03 | |  | | 0.01 | 0.05 |
| Hexadecenoic acid TMS - isomer 1 (C20) | Carboxylic acid | 0.03 | 0.1 |  | 0.01 | 0.04 |  | 0 | 0 | |  | | 0.01 | 0.06 |
| Hexadecenoic acid TMS - isomer 2 (C21) | Carboxylic acid | 0 | 0 |  | 0.02 | 0.07 |  | 0 | 0 | |  | | 0.01 | 0.04 |
| Heptadecanoic acid, trimethylsilyl ester (C25) | Carboxylic acid | 0 | 0 |  | 0.03 | 0.1 |  | 0 | 0 | |  | | 0.01 | 0.06 |
| 1-Octadecanol, O-TMS (C27) | Alcohol | 0 | 0 |  | 0.06 | 0.11 |  | 0.01 | 0.03 | |  | | 0.03 | 0.08 |
| 9,12-Octadecadienoic acid (Z,Z)-, trimethylsilyl ester (C30) | Carboxylic acid | 0.02 | 0.06 |  | 0.53 | 0.92 |  | 0.34 | 0.77 | |  | | 0.32 | 0.73 |
| Octadecenoic acid TMS - Isomer 1 (C31) | Carboxylic acid | 0 | 0 |  | 0.31 | 0.73 |  | 0.11 | 0.23 | |  | | 0.16 | 0.5 |
| Octadecenoic acid TMS- Isomer 2 (C32) | Carboxylic acid | 0 | 0 |  | 0.11 | 0.3 |  | 0.02 | 0.09 | |  | | 0.05 | 0.2 |
| Octadecanoic acid, trimethylsilyl ester (C33) | Carboxylic acid | 0.22 | 0.19 |  | 0.67 | 1.04 |  | 0.38 | 0.61 | |  | | 0.46 | 0.77 |
| Eicosanoic acid, trimethylsilyl ester (C38) | Carboxylic acid | 0.41 | 0.27 |  | 1.06 | 0.7 |  | 0.4 | 0.4 | |  | | 0.68 | 0.61 |
| Docosanoic acid, trimethylsilyl ester (C43) | Carboxylic acid | 0.08 | 0.11 |  | 0.3 | 0.24 |  | 0.05 | 0.09 | |  | | 0.16 | 0.21 |
| Squalene (C50) | Alkene | 0.76 | 2.45 |  | 0.01 | 0.04 |  | 0 | 0 | |  | | 0.24 | 1.38 |
| 1-Hexacosanol, TMS (C54) | Alcohol | 0.07 | 0.17 |  | 0 | 0.01 |  | 0 | 0 | |  | | 0.02 | 0.1 |
| Tocopherol-γ-tms-derivative or Tocopherol-β-tms-derivative (C55) | Tocopherols | 0.01 | 0.03 |  | 0.01 | 0.03 |  | 0.04 | 0.11 | |  | | 0.02 | 0.06 |
| (+)-α-Tocopherol, O-trimethylsilyl- (C62) | Tocopherols | 5.23 | 9.95 |  | 26.9 | 15.23 |  | 18.52 | 9.31 | |  | | 17.92 | 15.23 |
| Cholesterol TMS (C63) | Steroid | 25.89 | 13.22 |  | 21.38 | 12.23 |  | 18.17 | 7.42 | |  | | 21.93 | 11.64 |
| 5α-cholestan-3β-ol, TMS (C64) | Steroid | 0.94 | 0.62 |  | 1.85 | 1.46 |  | 5.6 | 9.92 | |  | | 2.57 | 5.4 |
| Cholecalciferol, TMS (C65) | Steroid | 0.83 | 1.49 |  | 5.13 | 2.88 |  | 2.46 | 1.92 | |  | | 3.08 | 2.92 |
| (22E)-3-[(Trimethylsilyl)oxy]ergosta-5,7,22-triene (C67) | Steroid | 1.47 | 1.8 |  | 4.82 | 3.91 |  | 3.57 | 1.98 | |  | | 3.44 | 3.2 |
| Campesterol, TMS (C68) | Steroid | 4.38 | 3.66 |  | 6.13 | 4.25 |  | 5.02 | 2.08 | |  | | 5.29 | 3.61 |
| 3-[(Trimethylsilyl)oxy]ergost-7-ene (C69) | Steroid | 0.29 | 0.56 |  | 0 | 0 |  | 0 | 0 | |  | | 0.09 | 0.33 |
| β-Sitosterol TMS (C71) | Steroid | 1.98 | 1.42 |  | 3.01 | 1.64 |  | 2.14 | 1.16 | |  | | 2.46 | 1.51 |
| Stigmastanol, TMS (C72) | Steroid | 0.08 | 0.16 |  | 0.7 | 0.91 |  | 1.16 | 1.95 | |  | | 0.63 | 1.21 |

Supplementary Table S2. Likelihood ratio tests comparing two candidate models for chemical diversity (Shannon index), body condition index and five habitat-related compounds. The null model (without habitat as a fixed factor) is compared to the full model (includes habitat as fixed factor). In all cases, except for body condition index, the full model (with habitat) received higher support than the null model. Statistically significant values marked with an asterisk.

| **Shannon index** | |  |  |  |  |  |  |  |  |
| --- | --- | --- | --- | --- | --- | --- | --- | --- | --- |
|  |  | *Model* | *df* | *AIC* | *BIC* | *logLik* | *Test* | *L.Ratio* | *p-value* |
|  | Null model | 1 | 3 | 26.51 | 31.93 | -10.26 |  |  |  |
|  | Full model | 2 | 5 | 18.37 | 27.41 | -4.19 | 1 vs 2 | 12.14 | 0.002* |
|  |  |  |  |  |  |  |  |  |  |
| **Body condition index** | |  |  |  |  |  |  |  |  |
|  |  | *Model* | *df* | *AIC* | *BIC* | *logLik* | *Test* | *L.Ratio* | *p-value* |
|  | Null model | 1 | 3 | -41.87 | -36.65 | 23.93 |  |  |  |
|  | Full model | 2 | 5 | -43.06 | -34.37 | 26.53 | 1 vs 2 | 5.20 | 0.074 |
|  |  |  |  |  |  |  |  |  |  |
| **Dodecanoic acid (C6)** | | |  |  |  |  |  |  |  |
|  |  | *Model* | *df* | *AIC* | *BIC* | *logLik* | *Test* | *L.Ratio* | *p-value* |
|  | Null model | 1 | 3 | -19.46 | -14.04 | 12.73 |  |  |  |
|  | Full model | 2 | 5 | -30.82 | -21.79 | 20.41 | 1 vs 2 | 15.37 | 0.000* |
|  |  |  |  |  |  |  |  |  |  |
| **Tetradecanoic acid (C10)** | | |  |  |  |  |  |  |  |
|  |  | *Model* | *df* | *AIC* | *BIC* | *logLik* | *Test* | *L.Ratio* | *p-value* |
|  | Null model | 1 | 3 | 75.32 | 80.74 | -34.66 |  |  |  |
|  | Full model | 2 | 5 | 63.69 | 72.72 | -26.84 | 1 vs 2 | 15.64 | 0.000* |
|  |  |  |  |  |  |  |  |  |  |
| **n-Pentadecanoic acid (C14)** | | |  |  |  |  |  |  |  |
|  |  | *Model* | *df* | *AIC* | *BIC* | *logLik* | *Test* | *L.Ratio* | *p-value* |
|  | Null model | 1 | 3 | -9.09 | -3.67 | 7.54 |  |  |  |
|  | Full model | 2 | 5 | -17.73 | -8.70 | 13.87 | 1 vs 2 | 12.65 | 0.002* |
|  |  |  |  |  |  |  |  |  |  |
| **α-Tocopherol (C62)** | |  |  |  |  |  |  |  |  |
|  |  | *Model* | *df* | *AIC* | *BIC* | *logLik* | *Test* | *L.Ratio* | *p-value* |
|  | Null model | 1 | 3 | 143.21 | 148.63 | -68.60 |  |  |  |
|  | Full model | 2 | 5 | 132.24 | 141.28 | -61.12 | 1 vs 2 | 14.96 | 0.001* |
|  |  |  |  |  |  |  |  |  |  |
| **Cholecalciferol (C65)** | | |  |  |  |  |  |  |  |
|  |  | *Model* | *df* | *AIC* | *BIC* | *logLik* | *Test* | *L.Ratio* | *p-value* |
|  | Null model | 1 | 3 | 102.54 | 107.96 | -48.27 |  |  |  |
|  | Full model | 2 | 5 | 92.60 | 101.63 | -41.30 | 1 vs 2 | 13.94 | 0.001* |

Supplementary Table S3. Multilevel pairwise comparisons (function: pairwiseAdonis) across localities for chemical composition of sand lizard FGs. Log transformed normalized peak ratio values were used as input. Multilevel pairwise comparisons adjusted P values are shown (p.adjust ='fdr') for each locality (habitat in brackets): Puszcza Niepołomicka (Rural); Krzemionki (Urban); Bodzów (Suburban); Bonarka (Urban); Mydlniki (Suburban); Młynka (Rural).

| **Comparisons** | **Df** | **SumsOfSqs** | **F.Model** | **R2** | **p.value** | **p.adjusted** |
| --- | --- | --- | --- | --- | --- | --- |
| Bodzów (Suburban) vs Bonarka (Urban) | 1 | 0.107 | 3.543 | 0.157 | 0.004 | 0.011 |
| Bodzów (Suburban) vs Mydlniki (Suburban) | 1 | 0.037 | 1.192 | 0.066 | 0.330 | 0.330 |
| Bodzów (Suburban) vs Puszcza Niepołomicka (Rural) | 1 | 0.428 | 10.197 | 0.338 | 0.001 | 0.008 |
| Bodzów (Suburban) vs Krzemionki (Urban) | 1 | 0.128 | 4.405 | 0.206 | 0.005 | 0.011 |
| Bodzów (Suburban) vs Mlynka (Rural) | 1 | 0.760 | 21.918 | 0.549 | 0.001 | 0.008 |
| Bonarka (Urban) vs Mydlniki (Suburban) | 1 | 0.071 | 2.183 | 0.179 | 0.088 | 0.094 |
| Bonarka (Urban) vs Puszcza Niepołomicka (Rural) | 1 | 0.192 | 3.930 | 0.232 | 0.005 | 0.011 |
| Bonarka (Urban) vs Krzemionki (Urban) | 1 | 0.072 | 2.453 | 0.197 | 0.077 | 0.090 |
| Bonarka (Urban) vs Młynka (Rural) | 1 | 0.413 | 10.783 | 0.495 | 0.003 | 0.011 |
| Mydlniki (Suburban) vs Puszcza Niepołomicka (Rural) | 1 | 0.196 | 3.681 | 0.251 | 0.019 | 0.029 |
| Mydlniki (Suburban) vs Krzemionki (Urban) | 1 | 0.069 | 2.267 | 0.221 | 0.078 | 0.090 |
| Mydlniki (Suburban) vs Młynka (Rural) | 1 | 0.426 | 10.283 | 0.533 | 0.005 | 0.011 |
| Puszcza Niepołomicka (Rural) vs Krzemionki (Urban) | 1 | 0.137 | 2.708 | 0.198 | 0.042 | 0.057 |
| Puszcza Niepołomicka (Rural) vs Młynka (Rural) | 1 | 0.209 | 3.662 | 0.234 | 0.007 | 0.012 |
| Krzemionki (Urban) vs Młynka (Rural) | 1 | 0.354 | 9.344 | 0.509 | 0.006 | 0.011 |

Supplementary Table S4. Spearman correlations of the amounts of five habitat-related compounds and BCI. Compound id’s as in supplementary Table S1.

|  |  | **Spearman correlation** | |  |
| --- | --- | --- | --- | --- |
|  |  | *rho* | *p* |  |
| **Rural** | **id** |  |  |  |
|  | C6 | -0.25 | 0.38 | * |
|  | C10 | -0.42 | 0.14 |  |
|  | C14 | -0.21 | 0.47 | * |
|  | C62 | -0.42 | 0.14 | * |
|  | C65 | -0.19 | 0.51 | * |
| **Suburban** |  |  |  |  |
|  | C6 | 0.05 | 0.85 |  |
|  | C10 | 0.23 | 0.39 |  |
|  | C14 | 0.05 | 0.85 | * |
|  | C62 | -0.23 | 0.39 |  |
|  | C65 | -0.11 | 0.7 |  |
| **Urban** |  |  |  |  |
|  | C6 | 0.1 | 0.76 | * |
|  | C10 | 0.13 | 0.68 |  |
|  | C14 | -0.06 | 0.87 | * |
|  | C62 | -0.36 | 0.26 |  |
|  | C65 | -0.4 | 0.2 | * |
| *cannot compute exact p-value with ties | | | |  |

**Supplementary figures**

Supplementary Figure S1. Visual representation of chemical signal composition of femoral gland secretions of 39 samples of male lizards in a Nonmetric Multidimensional Scaling (NMDS) according to habitat. Samples collected in 2021 (locality “Mlynka”) were excluded from this analysis. The ordination plot includes 95% confidence ellipses around group centroids (function: ordiellipse, kind = “se”, conf = 0.95). Each color represents one habitat. A distance matrix on log transformed normalized peak ratios was used as input.


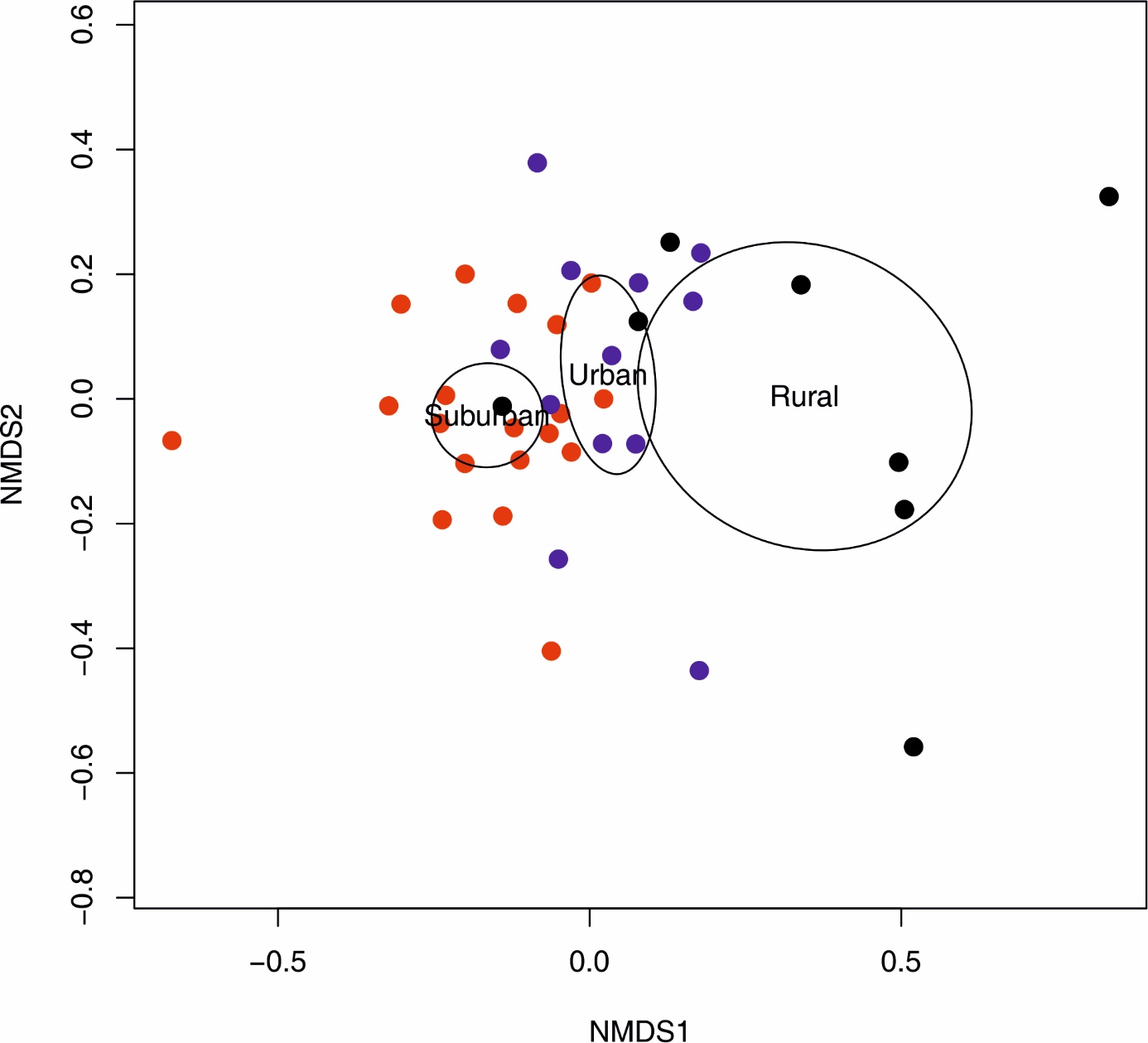


Supplementary Figure S2. Visual representation of chemical signal composition of femoral gland secretions of 45 samples of male lizards in a Nonmetric Multidimensional Scaling (NMDS) according to locality of origin (six localities in total) using a distance matrix of log transformed normalized peak ratio as input. The ordination plot includes 95% confidence ellipses around group centroids (function: ordiellipse, kind = “se”, conf = 0.95). Each color represents one locality: Puszcza Niepołomicka (Rural); Krzemionki (Urban); Bodzów (Suburban); Bonarka (Urban); Mydlniki (Suburban); Młynka (Rural).


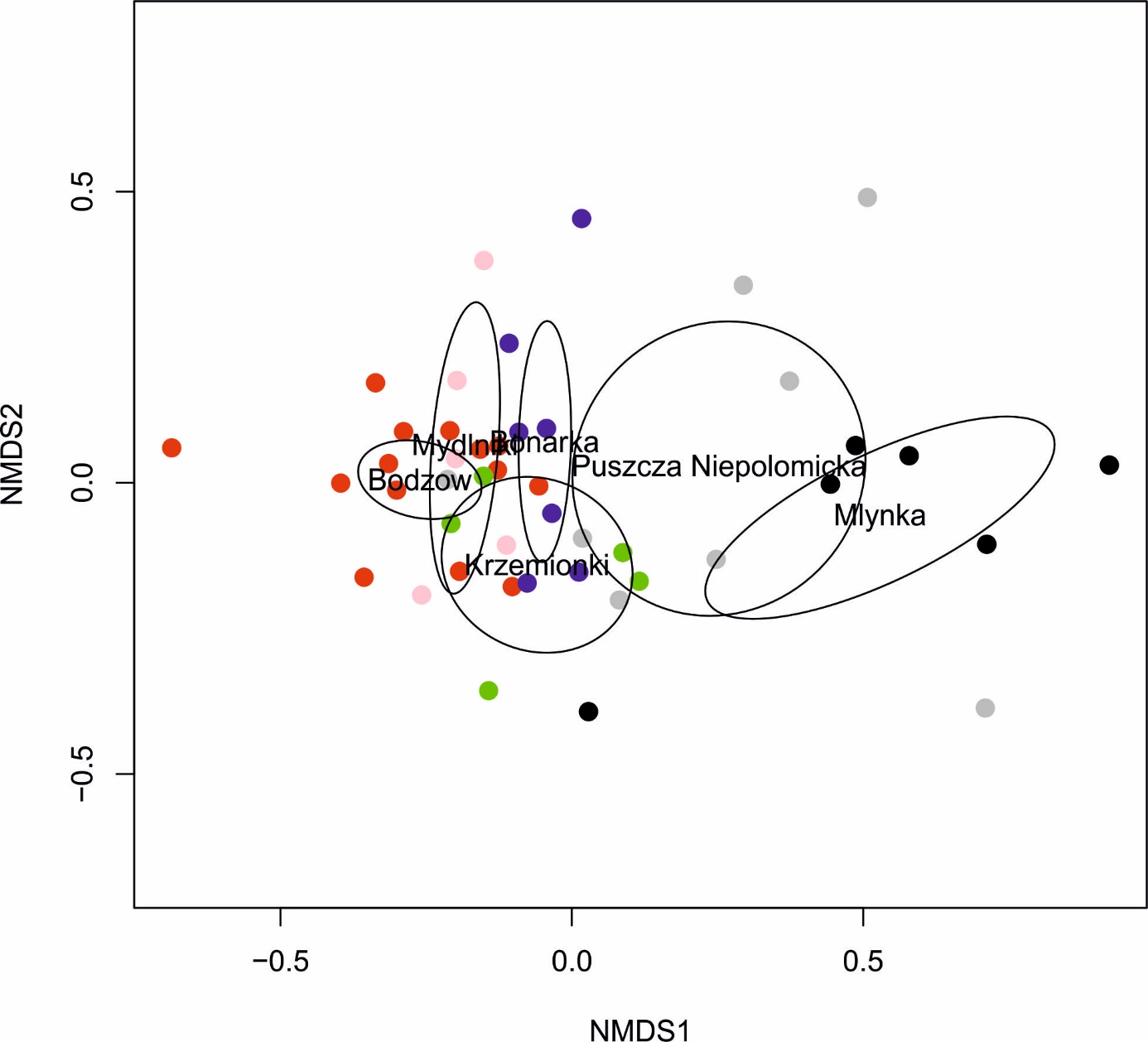


Supplementary Figure S3. Boxplot of Body Condition Index (BCI) across the six localities sampled in this study. Colors are shown for each habitat type according to the legend.


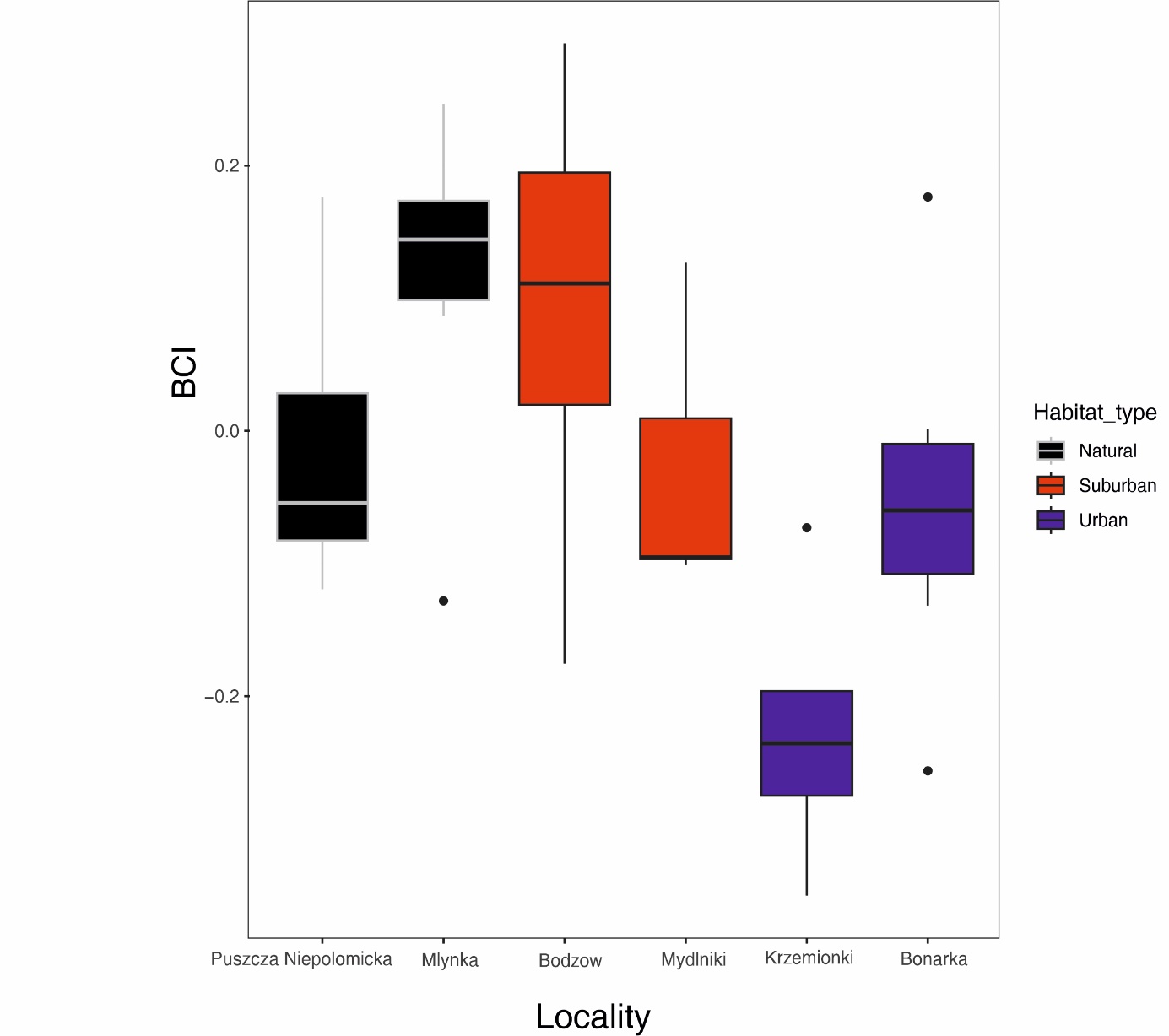

Supplement: Supplementary file 1 — Supplementary Information. [file 41598_2025_90393_MOESM1_ESM.docx]
